# Rapidly Enlarging Forearm Mass in a 74-Year-Old Male: A Case of Merkel Cell Carcinoma Presenting as a Soft Tissue Tumor

**DOI:** 10.7759/cureus.114030

**Published:** 2026-08-05

**Authors:** Sierra A Ziegler, Corey R Zimmer, Brian Ziegler

**Affiliations:** 1 Medicine, Lake Erie College of Osteopathic Medicine, Erie, USA; 2 Orthopaedic Surgery, The Orthopaedic Institute, Rockledge, USA

**Keywords:** forearm neoplasm, merkel cell carcinoma (mcc), neuroendocrine tumor, orthopedic oncology, soft tissue mass

## Abstract

Merkel cell carcinoma (MCC) is a rare, aggressive cutaneous neuroendocrine malignancy that most commonly presents in elderly patients as a rapidly growing lesion on sun-exposed skin. Because of its nonspecific clinical appearance, it can mimic a variety of benign and malignant soft tissue conditions, making diagnosis challenging. We report a rare presentation of MCC in a 74-year-old male who presented to the orthopedic clinic with a progressively enlarging soft tissue mass on the dorsoradial aspect of his right forearm. The patient reported a six-week history of swelling and constant radial-sided forearm pain that failed to improve with oral antibiotics and corticosteroids. Physical exam demonstrated a firm, deep subcutaneous mass with overlying mild warmth and flushing. Magnetic resonance imaging revealed a heterogeneous, enhancing soft tissue lesion concerning for soft tissue sarcoma, but final pathology confirmed MCC. This case highlights the diagnostic challenges of MCC and the importance of early recognition and tissue diagnosis in rapidly enlarging masses.

## Introduction

Merkel cell carcinoma (MCC) is a rare and aggressive cutaneous neuroendocrine malignancy that primarily affects older adults, particularly fair-skinned individuals with significant ultraviolet (UV) exposure, immunosuppression, or infection with Merkel cell polyomavirus [1,2]. Clinically, MCC typically presents as a rapidly enlarging, painless, firm, red to violaceous cutaneous nodule on sun-exposed skin, most commonly involving the head and neck, followed by the upper and lower extremities [2-7]. Because of its nonspecific clinical appearance, MCC may be mistaken for benign inflammatory lesions, infectious processes, cysts, or other malignant soft tissue tumors, including soft tissue sarcoma, often delaying diagnosis [3,6,8,9]. While imaging modalities such as magnetic resonance imaging (MRI) are useful for characterizing the extent of a soft tissue mass and narrowing the differential diagnosis, definitive diagnosis requires histopathologic evaluation with appropriate immunohistochemical staining [8,9]. This case illustrates an uncommon orthopedic presentation of MCC as a rapidly enlarging deep subcutaneous forearm mass initially suspected to represent a soft tissue sarcoma, highlighting the importance of maintaining a broad differential diagnosis and obtaining timely tissue biopsy for atypical soft tissue lesions.

## Case presentation

A 74-year-old left-hand dominant male with a past medical history of carcinoma of the prostate, status post remote prostatectomy, presented to the orthopedic clinic with a six-week history of right forearm pain and swelling. He reported initial evaluation by his primary care physician assistant, where he was presumed to have a soft tissue abscess and was treated with oral trimethoprim-sulfamethoxazole, followed by a course of prednisone when it did not improve or resolve. Ultimately, he was referred for orthopedic evaluation.

The patient described an insidious onset of a rapidly enlarging soft tissue mass over the dorsoradial aspect of the right distal forearm associated with constant radial-sided wrist pain rated 3/10. He denied fever, chills, night sweats, weight loss, trauma, or open wounds.

**Figure 1:**
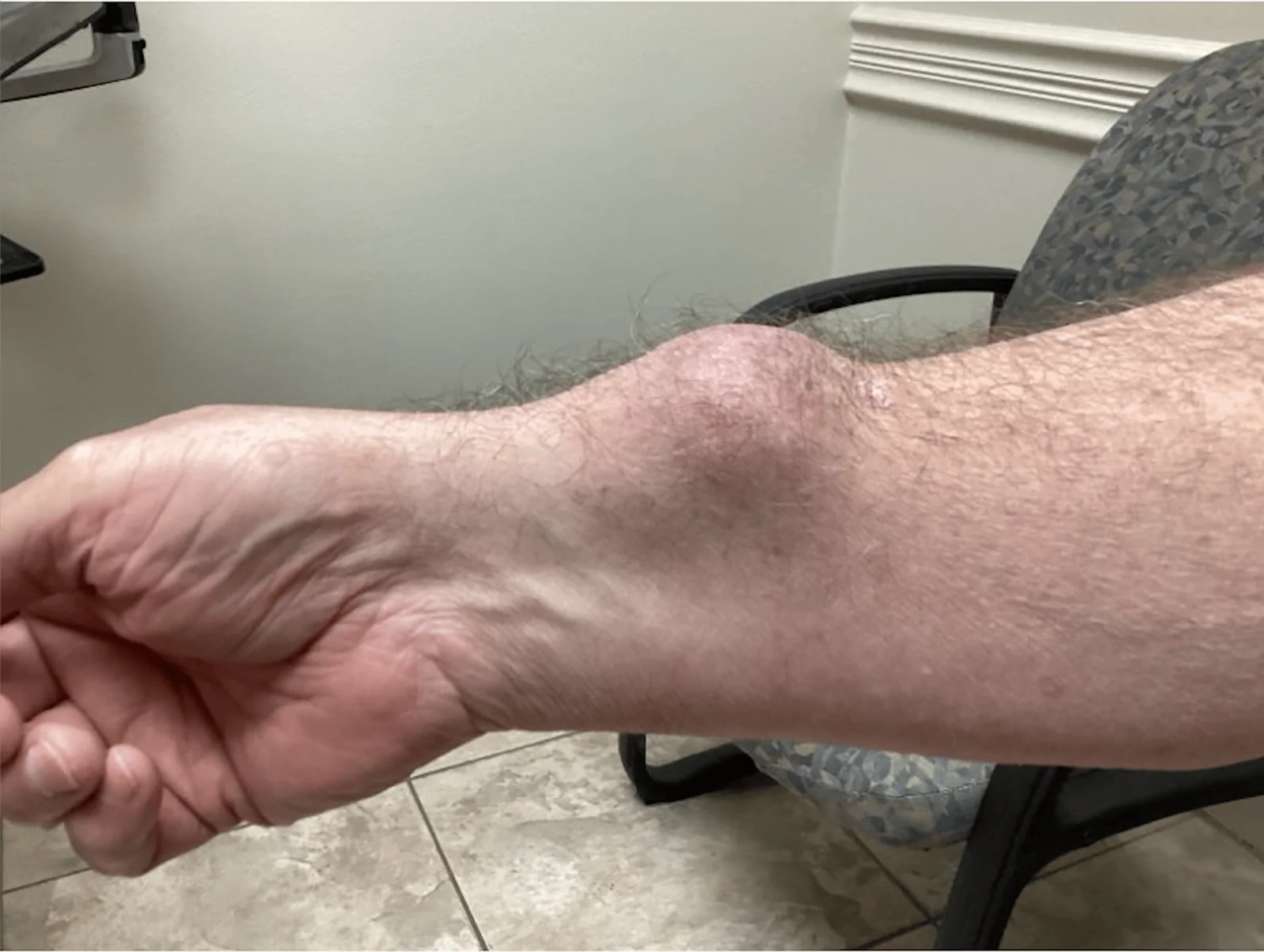
Initial clinical presentation demonstrating a firm subcutaneous mass over the dorsoradial aspect of the right distal forearm measuring approximately 5 x 5 cm. The lesion demonstrated mild overlying erythema and warmth without fluctuance or drainage.

Physical exam revealed a 5 x 5 cm firm, deep subcutaneous mass on the dorsoradial forearm, with mild warmth and flushing of the overlying skin (Figure 1).

No fluctuance or drainage was present, and the mass was moderately tender to palpation. The elbow, wrist, and finger range of motion were full and painless, and the motor and sensory exams were normal. No clinically apparent primary cutaneous lesion separate from the mass was identified.

Plain radiographs of the forearm and wrist revealed severe thumb carpometacarpal degenerative joint disease with dorsoradial forearm soft tissue swelling, but no osseous abnormality.

**Figure 2:**
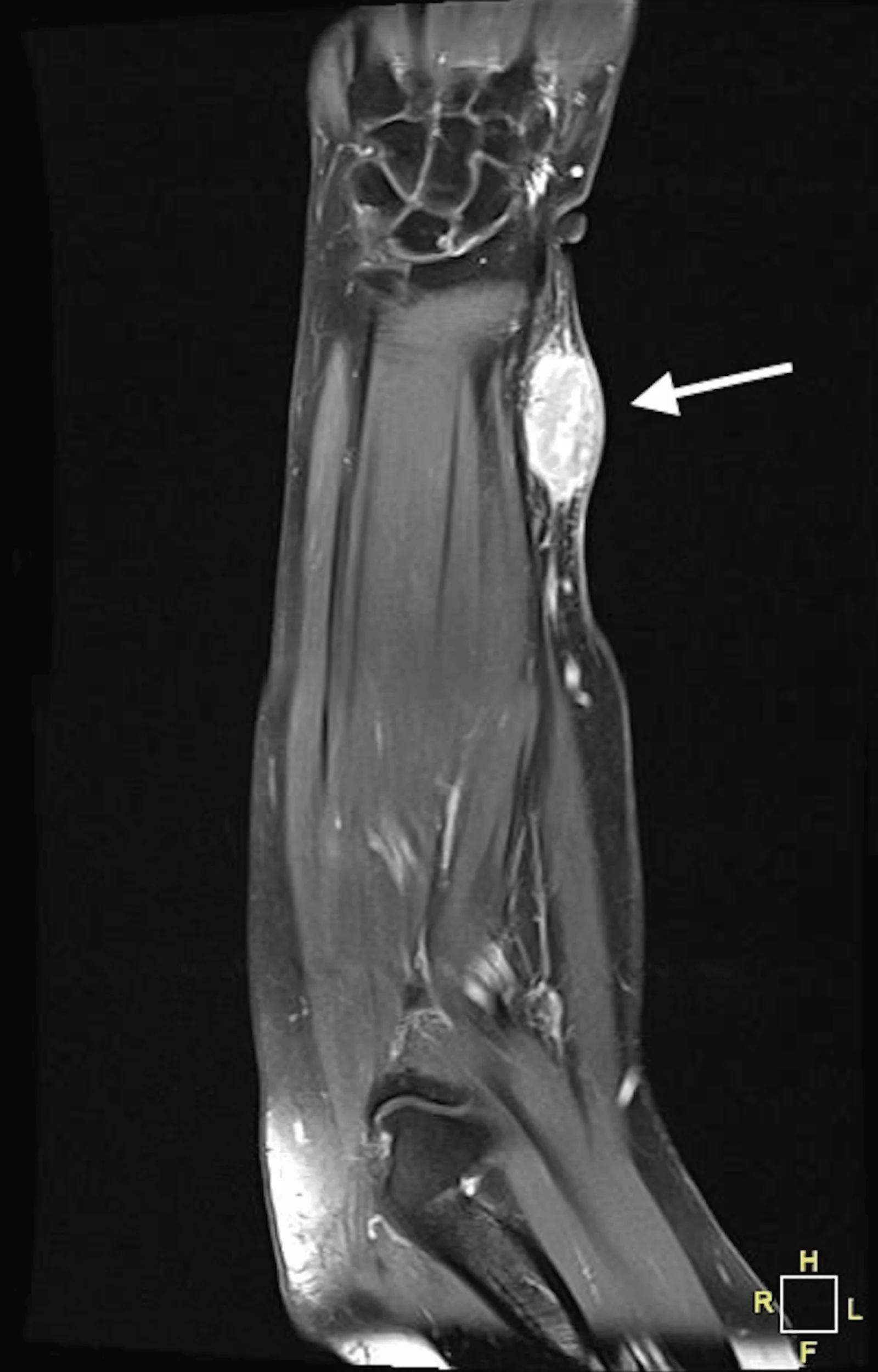
T1 coronal magnetic resonance image post IV gadolinium contrast of the right forearm showing a heterogeneous, lobular soft tissue mass in the subcutaneous fat, measuring 4.2 x 2.0 x 4.0 cm, with post-contrast enhancement most consistent with sarcoma. The lesion was confined to the subcutaneous tissues without involvement of the underlying muscle, tendon, or bone.

MRI of the wrist with and without intravenous (IV) gadolinium contrast (Figure 2) revealed a heterogeneous, lobular soft tissue mass within the subcutaneous fat, measuring 4.2 x 2.0 x 4.0 cm, with T2 hyperintensity and post-contrast enhancement most consistent with sarcoma [8,10]. No invasion of underlying muscles, tendons, or bones was identified.

Laboratory studies revealed alkaline phosphatase of 50 IU/L (44-121 IU/L), erythrocyte sedimentation rate of 8 mm/hr (0-30 mm/hr), and C-reactive protein of 7 mg/L (0-10mg/L), which were within normal limits.

An ultrasound-guided core needle biopsy of the mass was performed, with findings of fibrovascular tissue fragments extensively involved by high-grade poorly differentiated malignancy, having features of high-grade neuroendocrine carcinoma, most consistent with MCC [1,3,4,11]. Immunohistochemical stains showed positive keratin AE1/AE3, keratin 20, synaptophysin, and chromogranin staining. Keratin 20 showed focal perinuclear dot-like accentuation. The tumor was negative for prostate-specific antigen (PSA), CD20, CD19, CD3, S100, and melan-A.

Following histopathologic diagnosis, a nuclear medicine bone scan demonstrated only degenerative uptake in both triscaphe joints without evidence of osseous metastatic disease. Subsequent fluorodeoxyglucose (FDG) PET demonstrated an FDG-avid right radial forearm mass measuring 3.7 x 2.9 cm with a maximum standardized uptake value (SUVmax) of 7.32. No additional FDG-avid lesions or evidence of regional or distant metastatic disease were identified. Based on the imaging findings and biopsy results, the lesion was diagnosed as localized stage IIA MCC according to the 8th Edition of the American Joint Committee on Cancer (AJCC) classification for MCC [12].

**Figure 3:**
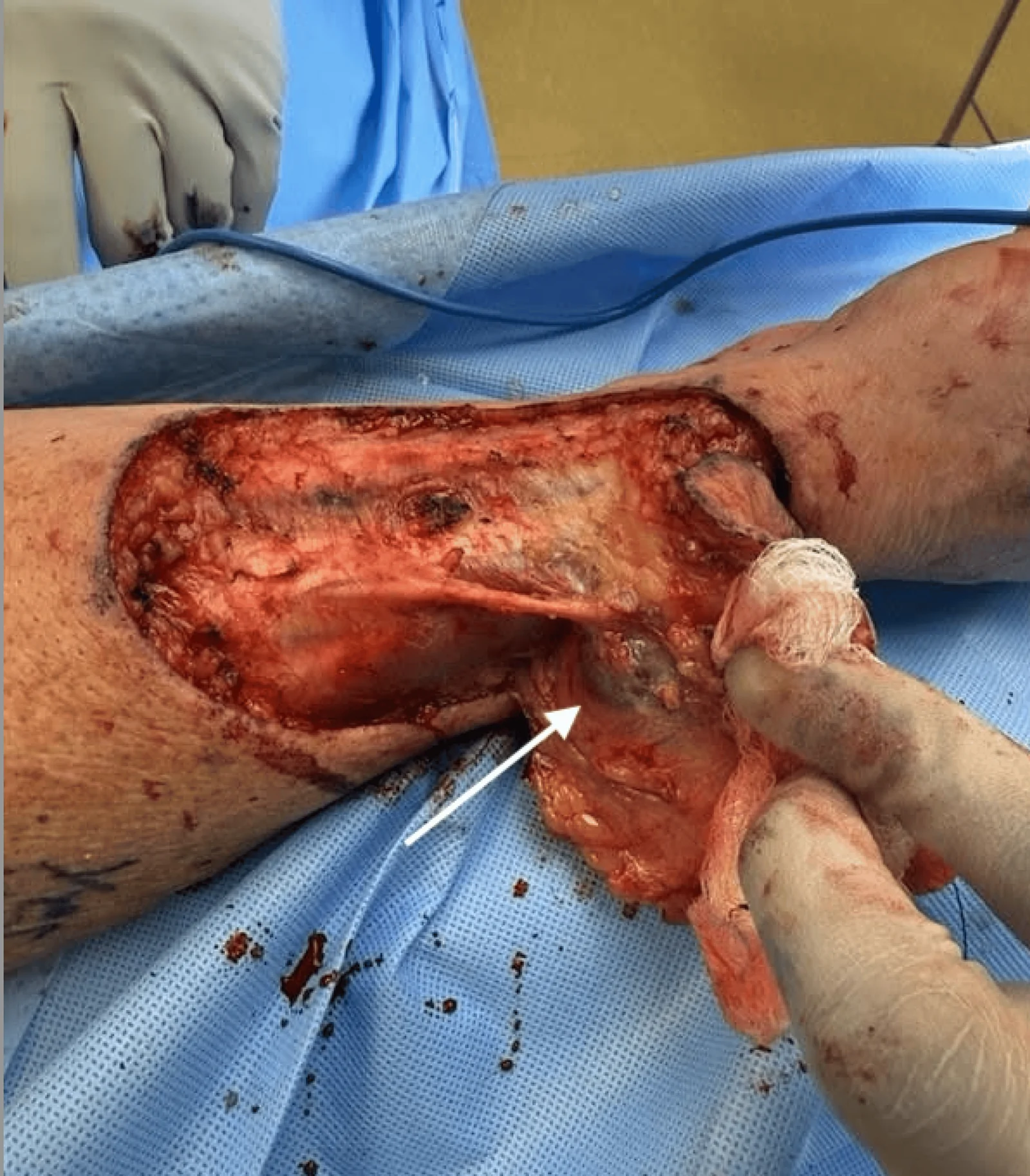
Intraoperative photograph demonstrating radical excision of the Merkel cell carcinoma from the dorsoradial forearm to the level of the muscular fascia.

The patient subsequently underwent radical excision of the tumor down to the level of the muscular fascia (Figure 3), and sentinel lymph node mapping and biopsy of six lymph nodes. The wound was managed with an Integra Bilayer membrane dressing [13,14]. Gross pathological examination demonstrated a 9.5 x 7.5 x 2.0 cm skin and soft tissue specimen containing a 4.2 x 3.5 x 2.5 cm encapsulated multinodular subcutaneous mass with a glistening pink to violaceous cut surface. The tumor was moderately vascular with dense fibrous septa and extended to within less than 1 mm of the deep surgical margin while remaining negative at the inked margin. The closest peripheral margin measured 1.5 cm away. Immunostains showed that the malignant cells were positive for cytokeratin AE1/3, cytokeratin 20 with a perinuclear pattern, synaptophysin, and weakly positive for p63, and were negative for cytokeratin 5/6 and cytokeratin 7. This supports the diagnosis of MCC. Sentinel lymph node biopsy of six right axillary lymph nodes showed no evidence of metastatic disease (0/6), corresponding to a pathological stage of pT2N0.

Multidisciplinary consultation was obtained. The consensus opinion was to proceed with adjuvant radiation treatment because of the close deep surgical margin and the morbidity associated with obtaining a wider deep margin, which would have required sacrifice of forearm musculature [1-4,6]. The patient underwent split-thickness skin grafting one month post-tumor excision.

Two months post-tumor excision, the patient began a course of adjuvant external beam radiation treatment consisting of 60 Gy in 30 fractions using 6 MV photons delivered to the surgical bed.

**Figure 4:**
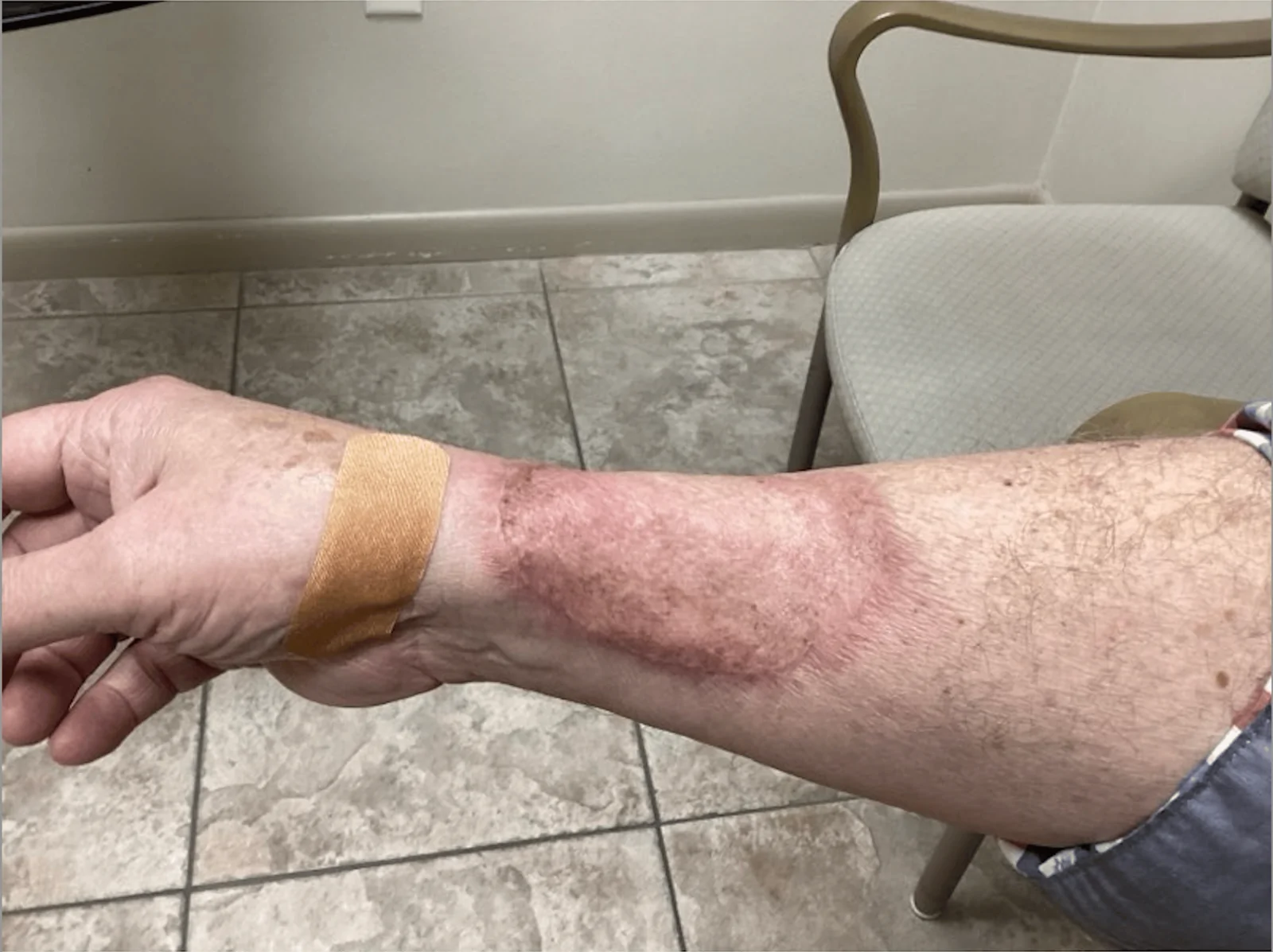
Clinical photograph obtained six months after tumor excision demonstrating a well-healed split-thickness skin graft following radical excision and completion of adjuvant radiotherapy. The patient had full forearm, wrist, and hand range of motion with an excellent functional outcome.

Six months post-tumor excision, the patient had a well-healed skin graft over the surgical bed (Figure 4) with minimal tenderness and full forearm, wrist, and hand motion. Mild paresthesias were present over the dorsoradial wrist, with otherwise normal motor and sensory function. Annual follow-up with MRI of the forearm, MRI of the brain, and computed tomography (CT) of the chest was recommended.

## Discussion

MCC is a rare but highly aggressive cutaneous neuroendocrine malignancy that most commonly affects elderly patients with significant UV exposure or other recognized risk factors, including immunosuppression and Merkel cell polyomavirus infection [1,2]. MCC most commonly arises on sun-exposed skin of the head and neck, followed by involvement of the trunk and extremities [2-7]. The distinguishing feature of this case was its presentation as a rapidly enlarging deep subcutaneous mass that clinically and radiographically mimicked a soft tissue sarcoma, leading to orthopedic referral rather than dermatologic evaluation [1,4,8,10].

The differential diagnosis of a rapidly enlarging upper extremity soft tissue mass is broad and includes soft tissue sarcoma, abscess, hematoma, lymphoma, metastatic disease, and other primary neuroendocrine malignancies [5,7]. In this patient, the lesion was initially treated as an infectious process with oral antibiotics and corticosteroids without improvement. Failure to respond to conservative therapy, continued rapid enlargement, and MRI findings concerning for malignancy appropriately prompted tissue diagnosis [1,4,11]. While MRI was valuable for defining the extent of the lesion and excluding invasion of adjacent muscles, tendons, and bone, it could not distinguish MCC from other malignant soft tissue tumors. This case emphasizes that biopsy remains essential for establishing a definitive diagnosis of atypical soft tissue masses.

Histopathologic examination remains the diagnostic gold standard for MCC. The biopsy demonstrated a high-grade poorly differentiated neuroendocrine carcinoma with immunohistochemical positivity for cytokeratin AE1/AE3, keratin 20 demonstrating the characteristic perinuclear dot-like staining pattern, synaptophysin, and chromogranin. The absence of staining for PSA, S100, melan-A, CD20, CD19, and CD3 helped exclude metastatic prostate carcinoma, melanoma, lymphoma, and other neoplasms, supporting the diagnosis of primary MCC [1,4,11]. The absence of additional FDG-avid lesions on PET and negative sentinel lymph node biopsy favored a primary subcutaneous MCC rather than metastatic neuroendocrine carcinoma.

Following multidisciplinary discussion, the patient underwent radical excision with sentinel lymph node biopsy. Although all surgical margins were negative, the tumor extended to within less than 1 mm of the deep margin. Obtaining a wider deep margin would have required sacrifice of the underlying forearm musculature with significant functional morbidity. Therefore, adjuvant radiotherapy was selected to improve local disease control while preserving upper extremity function. This treatment strategy is consistent with current recommendations supporting postoperative radiation for MCC with high-risk pathological features, including close surgical margins [1-4,6].

At six months following surgery, the patient demonstrated excellent functional recovery with a well-healed skin graft, full motion of the forearm, wrist, and hand, and no evidence of local recurrence. Nevertheless, MCC remains an aggressive malignancy with substantial risks of both local recurrence and distant metastasis, necessitating continued long-term surveillance with imaging and clinical follow-up [3,11,13-15].

This case report highlights the importance of maintaining a broad differential diagnosis for rapidly enlarging upper extremity soft tissue masses, particularly those that fail to respond to empiric treatment. Although uncommon, MCC should be considered in the evaluation of atypical soft tissue lesions because timely biopsy, accurate histopathologic diagnosis, appropriate staging, and multidisciplinary management are essential to optimizing oncologic and functional outcomes. A limitation of this report is the relatively short follow-up period, which precludes assessment of long-term recurrence or survival.

## Conclusions

This case illustrates the diagnostic challenges of MCC presenting as a rapidly enlarging deep subcutaneous forearm mass that clinically and radiographically mimicked a soft tissue sarcoma. Clinicians should maintain a broad differential diagnosis for enlarging soft tissue masses that fail to respond to empiric therapy, even when initial clinical findings suggest a benign or inflammatory process. Appropriate imaging may aid in lesion characterization; however, definitive diagnosis requires timely tissue biopsy with histopathologic and immunohistochemical evaluation. This case also highlights the importance of complete staging, multidisciplinary treatment planning, and appropriate postoperative surveillance in the management of MCC. This report reinforces the value of early recognition and tissue diagnosis in facilitating timely oncologic management of this rare malignancy.
